# Ag-Doped Phosphate Glass: Structure, Radio-Photoluminescence and Applications

**DOI:** 10.3390/ma19112204

**Published:** 2026-05-23

**Authors:** Meng Gu, Yaqi Peng, Xue Yang, Deyu Zhao, Yanshuo Han, Yihan Chen, Naixin Li, Kuan Ren, Jingtai Zhao, Qianli Li

**Affiliations:** 1 School of Materials Science and Engineering, Guilin University of Electronic Technology, Guilin 541004, China; gumeng1214@163.com (M.G.); zhaodeyu@shu.edu.cn (D.Z.); 2 State Key Laboratory of Advanced Refractories & School of Materials Science and Engineering, Shanghai University, Shanghai 200444, China; p1346471816@163.com (Y.P.); 1552500953@shu.edu.cn (X.Y.); 13403872261@shu.edu.cn (Y.H.); hanchenyi@shu.edu.cn (Y.C.); nxli725@icloud.com (N.L.); 3 National Key Laboratory of Plasma Physics, Laser Fusion Research Center, China Academy of Engineering Physics, Mianyang 621900, China; yunlongrk1990@sina.com

**Keywords:** Ag-doped phosphate glass, radio-photoluminescence, radiation dosimeter, X-ray imaging, high spatial resolution

## Abstract

Radiation detection technology is critical in medical diagnosis, high-energy physics experiments, nuclear environmental monitoring, and radiation safety protection. Its technological iteration stems from innovations in high-performance radiation detection materials. Traditional materials often have narrow dose–response intervals, insufficient high-precision measurement capability, low spatial resolution, and poor stability, failing to meet high-precision detection requirements. Ag-doped phosphate glass (Ag-PG), based on radio-photoluminescence (RPL), effectively addresses these limitations with its comprehensive advantages: high radiation sensitivity, a wide linear dose–response range, submicron spatial resolution for radiation imaging, write-erase-rewrite capability, and visualized dose monitoring potential, and it also boasts significant fundamental research value and engineering application prospects. Specifically, while existing RPL reviews mainly provide a comprehensive analysis from the perspective of RPL and present typical RPL material systems, this paper systematically analyzes the structural characteristics of the Ag-PG matrix and the coordination configuration and site occupation of Ag ions. It clarifies RPL luminescence properties, dose–response mechanisms, and the evolution of luminescence centers, while reviewing advancements in applications such as radiation dose detection and high-resolution X-ray imaging. By summarizing the current research status, technical advantages and existing challenges of Ag-PG, this study provides theoretical references and conceptual insights to promote breakthroughs in its fundamental research and practical applications in high-precision radiation dose detection, advanced medical imaging, micro-nano-scale radiation detection, and nuclear industry non-destructive testing.

## 1. Introduction

High-energy particles or radiation possess high energy and strong penetration capability. They can interact with matter to generate electron-hole pairs, thereby inducing significant radiation responses [1]. Based on this mechanism, radiation detection technologies have been widely applied in security inspection, radiation monitoring, medical diagnosis, and environmental monitoring. With the continuous development and iteration of radiation detection technologies, there is an increasing demand for detector materials with high sensitivity, high stability, and high spatial resolution [2,3]. Halide perovskite materials, as representative imaging materials, exhibit excellent performance in real-time X-ray imaging. However, their poor environmental stability and limited ability to store accumulated radiation dose hinder long-term applications [4,5]. Therefore, radiophotoluminescence (RPL) materials have become a research focus [6,7]. RPL refers to the formation of stable luminescence centers in materials after high-energy irradiation. Under ultraviolet excitation, these centers emit visible light, with the radiation energy stored in the form of electron–hole pairs. Compared with conventional optically stimulated luminescence (OSL) and thermally stimulated luminescence (TSL) materials, RPL materials exhibit higher sensitivity, more stable information storage, and re-readability [8,9,10,11]. The stored information can also be erased by high-temperature annealing, enabling reuse. OSL and TSL materials rely on the release of charge carriers from trap levels through thermal or optical stimulation [12]. Their readout process typically consumes the stored radiation information, leading to gradual signal attenuation or even irreversible loss [7]. This limits their application in high-precision repeated measurements. In contrast, RPL materials form stable defect centers after irradiation, such as Ag^0^ and Ag^2+^ centers in Ag-doped phosphate glass. Their readout process is based on photoexcitation rather than trap release, so the stored radiation information is not destroyed. This enables non-destructive readout and allows repeated measurements with nearly unchanged signal intensity [13].

**Figure 1:**
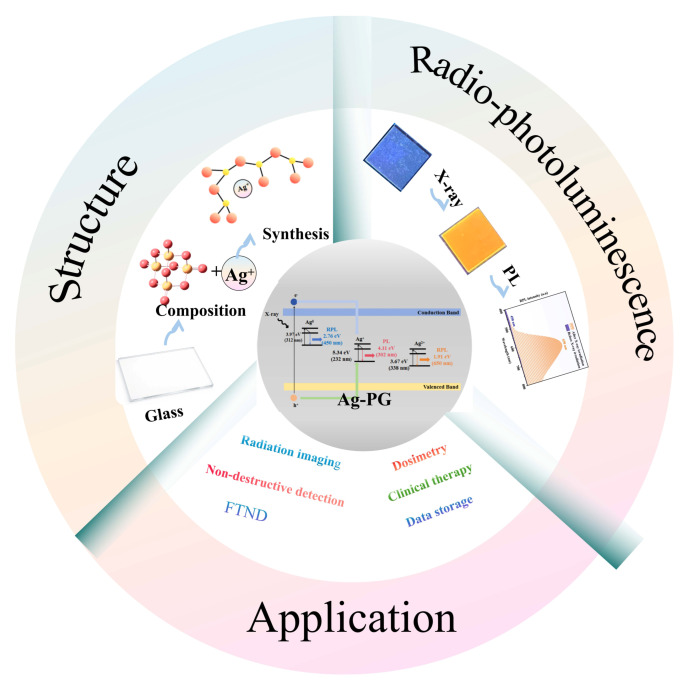
Schematic illustration of the structure, RPL mechanism, and typical applications of Ag-PG.

Ag-doped phosphate glass (Ag-PG) [14] is a typical RPL material. It uses a phosphate network as the host, with P–O–P linkages as the basic structural units. Silver ions are introduced to form luminescence centers, enabling radiation detection and imaging. As shown in Figure 1, Ag-PG exhibits excellent RPL performance. In recent studies, Ag-PG has demonstrated superior radiation sensitivity and high spatial resolution. It shows high sensitivity, a wide dose–response range, and submicron-resolution imaging capability. It also exhibits excellent thermal stability and tunable optical properties. These features make Ag-PG a promising candidate for high-dose radiation monitoring, submicron-resolution imaging, and rewritable radiation information storage. It provides a solid basis for the development of next-generation RPL materials [11].

Compared with Ag-PG, other reported RPL materials also show good radiation response, but clear gaps still remain in performance and applications. Among them, SrF_2_:Eu [15] is a representative RPL material. It exhibits excellent high-dose detection and imaging capability. The luminescent signal remains stable even above 5000 Gy, indicating strong radiation tolerance and high signal reliability. Its spatial resolution can reach 16.6 lp/mm [16]. However, it does not achieve the submicron resolution of Ag-PG. The Ca_2_BO_3_Cl:Eu [17] system shows a wide dose–response range of 1–3000 Gy and good linearity. Its borate chloride structure, composed of [BO_3_] units and Cl^−^ coordination, provides high chemical stability and mechanical strength. This ensures structural integrity and signal reproducibility under complex radiation conditions. However, its sensitivity and spatial resolution are still lower than those of Ag-PG. Silicate-based RPL materials, such as (Ba_1−x_Sr_x_)_2_SiO_4_:Eu, also exhibit good dose linearity and stable information storage. In the range of 0–60 Gy, the signal shows a linear dependence on dose. The spatial resolution is about 4 lp/mm. The radiation-induced signal can be completely erased under 420 nm laser irradiation, enabling reversible readout and repeated use [12]. However, its dose range, sensitivity, and spatial resolution are inferior to those of Ag-PG. Its thermal stability and optical tunability are also weaker.

The research on this type of RPL glass can be traced back to the mid-20th century. Schulman J. H. et al. first reported the radiophotoluminescence phenomenon in silver-activated phosphate glass in 1951 and provided an initial dose–response range of 0.1–10 Gy [18]. On this basis, the Japanese industry promoted the engineering development of this material system. Nippon Electric Glass developed the SAPANS series RPL glasses and carried out systematic studies [19,20]. Subsequently, AGC (originating from early research at Toshiba) introduced the FD-7 glass dosimeter, enabling the commercialization of Ag-PG [21]. With the suppression of background noise and improvements in fabrication processes, the dose–response range of Ag-PG has been extended to 10 μGy–10^6^ Gy. It has been widely applied in radiation imaging, neutron detection, fluorescent nuclear track detection, and medical irradiation [21]. At present, the most widely used Ag-PG system is commercial Ag-doped Na–Al phosphate glass (e.g., FD-7/GD450). Therefore, this work focuses on the structure and RPL properties of Na–Al/Ag phosphate glass. It also compares other Ag-PG systems, such as Li–Al/Ag compositions, to further demonstrate the broad application potential of Ag-PG in radiation detection.

This paper reviews the structure, physicochemical properties, RPL characteristics, and applications of Ag-PG, with particular emphasis on the differences in experimental results related to luminescence centers and fluorescence decay lifetimes. Finally, the advantages and limitations of Ag-PG in the RPL field are summarized, existing challenges and possible solutions are discussed, and future development prospects are outlined, providing insights for the advancement and application of Ag-PG in radiation detection and radiation imaging.

## 2. Preparation of Ag-PG

**Figure 2:**
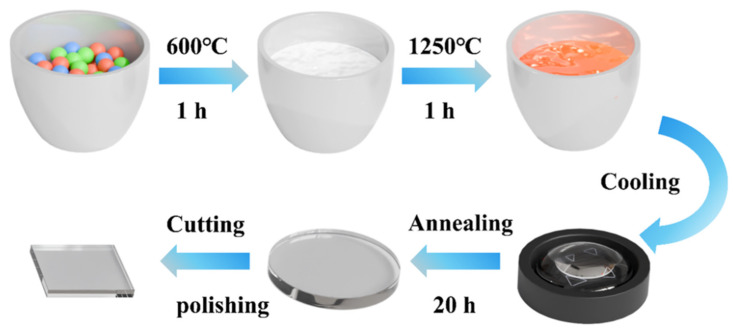
Schematic illustration of the preparation process of Ag-PG via high-temperature melt quenching.

Ag-PG is typically based on phosphate networks such as NaPO_3_ and Al(PO_3_)_3_, with Ag ions acting as luminescence centers. Its preparation has evolved from melt–slow cooling to high-temperature melt quenching (Figure 2), and these methods lead to different material properties [11,22].

Melt-slow cooling is closer to thermodynamic equilibrium and allows the glass structure to relax. However, it may cause local ordering or even crystallization, which reduces optical uniformity and affects the radiation response [23]. In contrast, melt quenching is more widely used. It rapidly freezes the melt structure, suppresses crystallization, and forms a stable amorphous network. The defects retained during quenching can be converted into luminescence centers (Ag^0^ and Ag^2+^) under irradiation, which helps improve RPL sensitivity and signal stability [24]. A post-annealing process is usually applied to release internal stress.

In terms of composition, monovalent cations such as Na^+^ and Li^+^ are important for stable RPL performance [25]. The Na–Al/Ag system has good glass-forming ability, high structural stability, and strong luminescence efficiency. It also shows reliable signal storage and is widely used in commercial dosimeters such as FD-7 and GD450. The Li–Al/Ag system can improve carrier mobility, is beneficial for high-energy particle detection and has good RPL properties [26]. However, the high field strength and mobility of Li^+^ may increase internal stress and reduce structural stability [27]. It can also decrease the stability of luminescence centers, leading to lower luminescence efficiency compared with the Na–Al system.

Furthermore, the performance of Ag-PG can be tuned through composition modification. For example, adding Na_2_O or NaCl can adjust radiation sensitivity [28], introducing SiO_2_ can improve thermal stability [6], B doping enables neutron detection [29], and using a borate glass matrix can enhance stability under high radiation doses [30].

## 3. Results

### 3.1. Properties of Ag-PG

#### 3.1.1. Structure of Phosphate Glass

**Figure 3:**
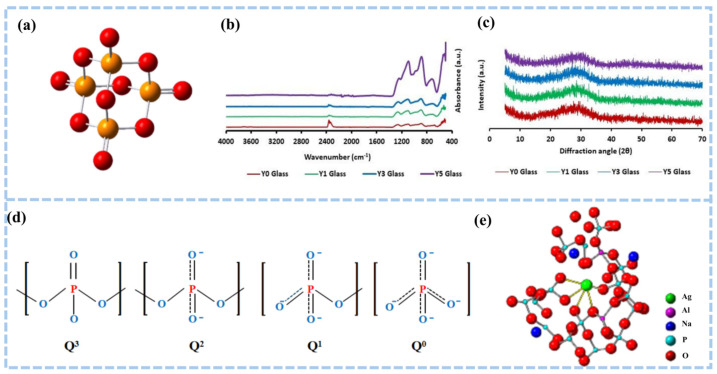
Basic structure of Ag-PG: (**a**) Molecular structural model of phosphate glass represented by P_4_O_10_ [36]; (**b**) FTIR spectrum identifying Q^1^ and Q^2^ structural units and P–O–P/P=O vibration bands [37]; (**c**) XRD pattern showing broad diffuse peaks and the amorphous nature of Ag-PG [37]; (**d**) Schematic of Q^n^ tetrahedral connection modes in phosphate glass networks; (**e**) Local atomic configuration around Ag^+^ cations in Ag-PG [39].

Phosphate glasses exhibit moderate glass transition temperature, low optical scattering, high refractive index and high thermal expansion coefficient [31,32,33,34]. They have wide application prospects in basic research and industrial production [34]. The structural unit of pure P_2_O_5_ glass is the P_4_O_10_ molecule [35]. Its network shows chain or layered structures, which are similar to the crystal structure of P_4_O_10_, but its stability is insufficient. The basic structural unit of phosphate glass is the [PO_4_]. The P=O double bond is the main reason for the chain or layered structure. Figure 3a shows the structure of P_4_O_10_ [36] (adapted from Molecular and biological activities of metal oxide-modified bioactive glass, by Tiama, T.M.; Elhaes, H.; Ibrahim, M.A.; et al., 2023. Licensed under CC BY 4.0.), the model molecule of phosphate glass. Its connection mode can be described by Q^n^ units (*n* = 0–3). It is more important to focus on the polymerization degree of the glass network, rather than repeating traditional structural classification. The polymerization degree directly determines the content of non-bridging oxygen (NBO). NBO plays a key role in the formation of luminescence centers. Changing the NBO ratio and local structure can affect the formation efficiency of Ag-PG defect centers, and thus control the RPL intensity [28].

Figure 3b,c only illustrate the intrinsic structural characteristics of phosphate glass [37] (Reprinted from Yttrium-doped phosphate-based glasses: structural and degradation analyses, by Arafat, A.; Samad, S.A.; Titman, J.J.; et al., 2020. Licensed under CC BY 4.0.). Figure 3b shows the FTIR spectrum of phosphate glass [33]. These findings prove the coexistence of Q^2^ and Q^1^ units in phosphate glass, indicating a partially depolymerized network structure. Such a structure achieves a balanced performance between network rigidity and flexibility, which is conducive to the stabilization of defect states and the capture of charge carriers. Accordingly, these structural characteristics are essential for the formation and stabilization of Ag^0^ and Ag^2+^ luminescence centers in Ag-PG. Figure 3c presents the XRD patterns of phosphate glass [33]. Only broad diffuse diffraction peaks can be observed in the spectrum, with no sharp crystalline diffraction peaks. This result verifies the amorphous structure of Ag-PG. The intrinsic network structure of phosphate glass plays a vital regulatory role in the RPL properties of Ag-PG. The glass network exhibits excellent flexibility and is susceptible to structural polymerization. It can produce abundant non-bridging oxygen and adjustable local coordination environments, creating favorable conditions for the generation of RPL luminescence centers. Meanwhile, the introduction of alkali metal ions (Na^+^, Li^+^) can effectively adjust the charge compensation mechanism of the system and facilitate the uniform distribution of multivalent silver luminescence centers. Moreover, phosphate glass has high density and effective atomic number. It can enhance the absorption of radiation energy and further improve the capture efficiency of electron-hole pairs. Also, with increasing Al_2_O_3_ content, the proportion of Q^3^ units increases rapidly, while the concentration of Q^2^ units also increases initially and then gradually decreases after reaching a maximum value. This variation may be attributed to the significant reduction of Q^1^ groups with increasing Al_2_O_3_ content, thereby confirming the formation of new P-O-Al structural units.

The incorporation of modifying oxides (e.g., Na_2_O) into amorphous P_2_O_5_ disrupts bridging oxygens (BO) and generates NBO. This depolymerization of the phosphate network is as follows [38]:

2Q^n^ + R_2_O → 2Q^n−1^ (1)

The variation law of Q^n^ tetrahedron composition is presented in Figure 3d.

In Ag-PG, Ag exists in a distorted local coordination environment with an average Ag-O bond length of ~2.5 Å, and the first coordination shell exhibits considerable disorder. This local structure is recognized as a distorted octahedral or trigonal bipyramidal geometry. The Ag-O coordination numbers determined by first-principles calculations and classical methods are 5.42 and 5.54–5.71, respectively. Figure 3e (adapted from Combinatorial characterization of metastable luminous silver cations, by Masai, H.; Koshimizu, M.; Kawamoto, H.; et al., 2024. Licensed under CC BY 4.0.) [39] illustrates the atomic configuration surrounding Ag^+^ cations in FD-7 glass, where Ag^+^ is primarily coordinated with oxygen atoms in Q^2^ phosphate units.

#### 3.1.2. RPL Properties of Ag-PG

As the most widely used RPL material, Ag-PG has been applied since the 20th century and has undergone more than 50 years of development; in recent years, it has attracted extensive attention in the fields of high-sensitivity, high-dose radiation detection and radiation imaging.

Upon exposure to high-energy rays (X-rays, β-rays) and high-energy particle irradiation, Ag-PG can emit orange light in the range of 600–700 nm under ultraviolet excitation at 310–385 nm. The typical Ag content in the Na–Al/Ag system of Ag-PG is approximately 0.1 wt%. At this doping concentration, silver ions are mainly dispersed in the glass network in the form of free Ag^+^. Under X-ray irradiation, abundant electron-hole pairs are generated. The released electrons are captured by Ag^+^ to form Ag^0^ luminescence centers. Meanwhile, the holes are initially trapped by PO_4_^3−^ to form phosphorus oxygen hole centers (PHOCs), which further migrate and combine with Ag^+^ to produce Ag^2+^ centers. Consequently, both Ag^0^ and Ag^2+^ act as the dominant luminescence centers of Ag-PG. Among them, Ag^2+^ contributes much stronger luminescence intensity, which fundamentally accounts for the orange emission of Ag-PG under excitation [40].

As summarized in Table 1, after X-ray irradiation, most Ag-PG samples exhibit two typical emission bands in the range of 400–800 nm under excitation at 310–390 nm. The blue emission peak near 450 nm corresponds to Ag^0^ centers, with characteristic excitation peaks around 270 nm and 340 nm. The broad orange emission band located at 630 ± 35 nm is assigned to Ag cluster-like centers, whose excitation bands are mainly concentrated in the 310–385 nm. In terms of the inconsistent emission peaks at 560 nm and 650 nm reported in previous studies, it should be emphasized that the Ag-PG systems adopted in this work are based on commercially standardized compositions. Their main network components and Ag doping concentrations are confined within a stable processing window, which greatly reduces the possibility of systematic peak shifts caused by intrinsic material composition. In this context, the obvious spectral difference between 560 nm and 650 nm is primarily attributed to the comprehensive response deviation of different spectral measurement systems, including the non-uniform wavelength dependence of grating diffraction efficiency, the uneven quantum efficiency distribution of detectors (PMT, CCD and other devices), and the differences in optical filters and integral modes in various experimental setups [40]. Without systematic correction of spectral response functions, these factors will induce significant offsets and apparent shape reconstruction of emission peaks.

In addition, Table 1 also confirms the existence of Ag ion polymers in the glass matrix, among which Ag^2+^ dimers serve as critical luminescence centers. The combination of Ag^+^ and Ag^0^ can form Ag^2+^ orange luminescence centers at 650 nm and further evolve into Ag_m_^n+^ polymer centers. In summary, the emission peak at 450 nm originates from Ag^0^ centers, while the luminescence signal near 650 nm is jointly contributed by Ag^2+^ and partial Ag_m_^n+^ polymer centers.

**Figure 4:**
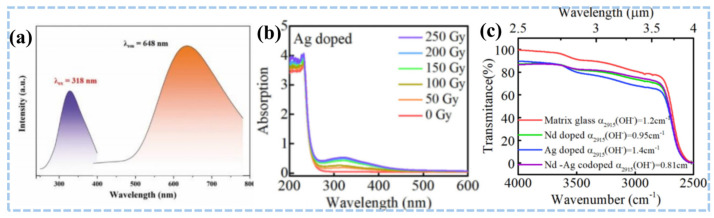
Basic RPL characteristics of Ag-PG: (**a**) The emission spectrum of luminescence centers [50]; (**b**) The absorption spectra before and after X-ray irradiation of luminescence centers [2]; (**c**) Optical transmittance spectra showing slight reduction after irradiation due to color center formation [2].

As shown in Figure 4a [50] (reprinted from Large-size flexible X-ray imaging and information encryption storage based on radio-photoluminescence, by Meng, G.; Li, N.; Wei, Y.; et al., 2026. Licensed under CC BY 4.0.), the emission spectrum of Ag-PG exhibits a blue emission peak at 450 nm attributed to Ag^0^ centers and an orange emission peak at 648 nm attributed to Ag^2+^ centers. As shown in Figure 4b [2], irradiated Ag-PG exhibits a distinct absorption peak centered at 310 nm, and its intensity gradually increases with the radiation dose, confirming the formation of UV-absorbing luminescence centers induced by irradiation. After Gaussian fitting, the induced absorption spectrum mainly contains three peaks: the band near 3.35 eV (370 nm) is assigned to Ag^0^ centers, while the bands at about 3.87 eV (320 nm) and 4.30 eV (288 nm) correspond to Ag^2+^ centers. Under irradiation, Ag^+^ ions capture electrons or holes to form stable Ag0 and Ag^2+^ luminescence centers, which can be eliminated only by annealing at 400 °C for 1 h. Figure 4c presents the infrared transmittance spectra of unirradiated Ag-PG [2]. Characteristic absorption bands near 3500 and 3000 cm^−1^ correspond to OH^−^ stretching vibrations. The OH^−^ absorption coefficient α(OH^−^) was calculated using α(OH^−^) = log(T_0_/T)/L. The α(OH^−^) values of the four samples are 1.2, 0.95, 1.4, and 0.81 cm^−1^, all below 1.5 cm^−1^, demonstrating efficient dehydration and negligible nonradiative quenching. Thus, the measured fluorescence lifetimes of Ag-related defect centers reliably reflect the intrinsic matrix effect. Figure 4b,c are reprinted from Silver-Neodymium Codoped Lithium Aluminum Metaphosphate Glasses for Radio-Photoluminescence Dosimeter, by Ma, X.; Cheng, J.; Fan, S.; et al., 2022. Licensed under CC BY 4.0.

**Figure 5:**
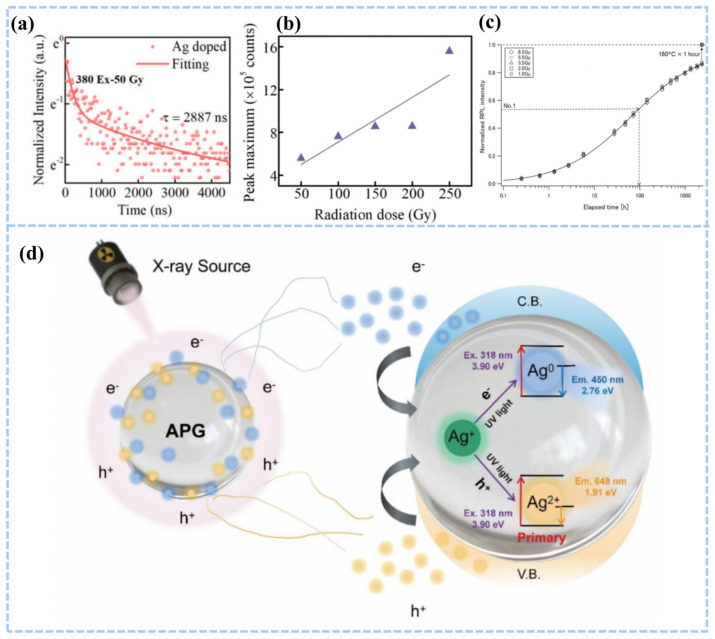
RPL properties and mechanism of Ag-PG: (**a**) Photoluminescence decay curves and lifetime evolution before and after irradiation [2]; (**b**) Linear dose–response relationship of orange RPL intensity [2]; (**c**) Build-up effect of RPL intensity and acceleration by thermal treatment [52]; (**d**) Schematic of the complete RPL process including carrier generation, trapping, and radiative transition [50].

Figure 5a shows the photoluminescence decay curves [2] (reprinted from Silver-Neodymium Codoped Lithium Aluminum Metaphosphate Glasses for Radio-Photoluminescence Dosimeter, by Ma, X.; Cheng, J.; Fan, S.; et al., 2022. Licensed under CC BY 4.0.). After irradiation, the decay lifetime corresponding to emissions at 650 nm is 2887 ns. Figure 5b presents the correlation between the maximum RPL intensity of Ag-related defect centers at 650 nm and the radiation dose in Ag-doped phosphate glasses [2] (reprinted from Silver-Neodymium Codoped Lithium Aluminum Metaphosphate Glasses for Radio-Photoluminescence Dosimeter, by Ma, X.; Cheng, J.; Fan, S.; et al., 2022. Licensed under CC BY 4.0.). As observed, the RPL peak intensity exhibits a weak dose-dependent fluorescence response. A good linear relationship between RPL intensity and dose is obtained in the range of 0–100 Gy. This linearity degrades at higher doses owing to the variation of the optical absorption coefficient, which requires signal correction in the high-dose region. The luminescence center intensity gradually increases with radiation exposure and finally reaches saturation under certain conditions, demonstrating a typical buildup effect [22]. At room temperature, this effect usually lasts for several hours [51] and can be accelerated by thermal treatment. As shown in Figure 5c, heating at 160 °C for 1 h promotes the saturation of RPL intensity [52] (reprinted from Estimation of elapsed time after an unnoticed radiation exposure using weathering-resistant RPL glass (SAPANS), by Yasuda, H.; Gonzales, C.A.B.; Aghabaklooei, S., 2023. Licensed under CC BY 4.0.). Figure 5d schematically illustrates the RPL process of Ag-PG [51] (reprinted from Large-size flexible X-ray imaging and information encryption storage based on radio-photoluminescence, by Meng, G.; Li, N.; Wei, Y.; et al., 2026. Licensed under CC BY 4.0.). When the incident ionizing radiation energy exceeds the bandgap of Ag-PG, electrons are excited from the valence band to the conduction band, generating electron-hole pairs. Electrons are captured by Ag^+^ to form Ag^0^, while holes are initially trapped by PO_4_^3−^ tetrahedra and then transferred to Ag^0^ through thermal release to form Ag^2+^. Under ultraviolet excitation, Ag^0^ and Ag^2+^ centers emit blue light (~450 nm) and orange light (~648 nm), respectively.

Table 2 shows that the decay times of Ag-PG are systematically analyzed. The decay time of Ag^0^ centers is generally shorter than 4.5 ns, typically ranging from 1 to 4.5 ns [47]. In contrast, the decay time of Ag^2+^ centers is more complex and strongly dependent on the local coordination environment and host glass structure. Previous studies have classified Ag^2+^ related emission lifetimes into three categories: a fast component (<2 μs), an intermediate component (2–10 μs), and a long-lived component (>20 μs) [42]. This result suggests that Ag^2+^ does not correspond to a single uniform luminescence center, but a group of structurally distinct species. Some studies assign a dominant lifetime of ~2 μs to isolated Ag^2+^ ions [53] ions in orange-emitting centers with stronger interactions with the glass network [44]. These differences are mainly caused by variations in glass composition, Ag concentration, defect structures, and local coordination symmetry, all of which significantly affect the radiative relaxation pathways of Ag^2+^ centers. In this work, Ag^2+^ emission is therefore regarded as a multi-component decay process rather than a single fixed lifetime.

### 3.2. Applications of Ag-PG

#### 3.2.1. Radiation Dose

A radiation dosimeter [56] is an instrument for measuring and evaluating ionizing radiation dose. Based on the response mode, dosimeters are divided into active [57] and passive types [58]. Active dosimeters convert radiation into electrical signal pulses, allowing the determination of radiation type and intensity from signal characteristics. In contrast, passive dosimeters store radiation information through interactions with sensitive media, which is then read out via physical stimulation; they are widely used for cumulative dose monitoring. According to luminescence mechanisms, luminescence-based dosimeters include thermally stimulated dosimeters (TLD), optically stimulated luminescence dosimeters (OSLD), and RPL glass dosimeters (RPLGD). In these systems, part of the absorbed radiation energy is stored in metastable states and later released as visible emission under external stimuli (e.g., heating or optical excitation). RPLGD is an integrating solid-state dosimeter based on Ag-PG. After ionizing radiation exposure, dose information is read out through orange emission under UV excitation.

Ag-PG exhibits favorable annealing recyclability. Annealing at 400 °C for 2 h can eliminate luminescence centers and restore it to the initial state, which enables recycled use without irreversible damage. Ag-PG exhibits excellent dose–response behavior at high doses. A linear response is observed from low to medium doses (<~4 kGy), followed by saturation and nonlinear/decay behavior in the ~4–10 kGy range due to competition between center formation and annihilation [49].

Ag-PG can be fabricated into a 3D-printed ear model [13]; its irradiated regions emit visible orange light under UV excitation, which enables spatial dose mapping. The RPL signal is visually detectable at doses as low as 500 mGy, and approximate dose distributions can be extracted via image processing even for complex geometries. Microspherical glass dosimeters (~0.1 mm) fabricated from Ag-PG can be dispersed over contaminated surfaces, and their spatial RPL distribution under UV illumination allows rapid visualization of radiation fields, providing a practical method for post-accident monitoring. Under a constant γ-ray dose, the RPL intensity of Ag-PG depends on the content of natural LiPO_3_, and it is found that the Ag-PG response to γ-rays degrades with increasing Li content [59]. In contrast, under a constant neutron fluence, the RPL intensity increases with increasing natural LiPO_3_ content, which significantly enhances the sensitivity to thermal neutrons. These results confirm the potential of Ag-PG for neutron detection [59]. Studies on the response of Ag-PG to α-particle irradiation demonstrate that its luminescence properties vary significantly with radiation types, including low-LET X-rays and high-LET α-particles. Such differences are reflected in the varied intensity ratio between blue and orange emission, which reveals the distinct LET dependence of the Ag-PG system. This finding provides a fundamental reference for the application of Ag-PG in radiation type discrimination and high-energy particle detection [60].

#### 3.2.2. Radiation Imaging

The mainstream X-ray imaging materials currently include films and scintillators. Although both can achieve micrometer-scale spatial resolution, each has inherent limitations. While films can provide high spatial resolution, their discontinuous structure arising from emulsion grains leads to a nonlinear response and inconsistent quantitative calibration, making precise data analysis difficult; moreover, the offline readout process further reduces experimental efficiency [61,62]. Scintillation materials generate visible light signals instantaneously under irradiation, which can be read out in real time through optical systems. The scintillation emission originates directly from radiation energy deposition within the material, and the emission distribution is closely correlated with the energy deposition region. However, in practical imaging processes, carrier diffusion within the scintillator and lateral propagation of scintillation photons are unavoidable, often leading to optical crosstalk between adjacent regions and thereby degrading spatial resolution [63]. Owing to its excellent radiation stability and visible emission under ultraviolet excitation, Ag-PG has been further explored for applications in radiation imaging. Compared with conventional scintillator materials, Ag-PG, as a typical storage-type RPL material, is limited by its inability to realize real-time X-ray imaging. This is because signal readout requires subsequent optical stimulation instead of immediate light emission upon irradiation, so it lacks the instantaneous response of scintillators. In addition, its imaging process depends on a scanning-based readout system. This increases system complexity and leads to poor temporal resolution, rendering Ag-PG unsuitable for dynamic or high-speed imaging applications.

Yucheng Li et al. [11] applied Ag-PG to biological imaging and industrial non-destructive testing. Specifically, Ag-PG was used to obtain the X-ray image of a small fish (~70 mm in length), where the head, thorax, and tail structures are clearly resolved. Benefiting from the excellent microscale spatial resolution of Ag-PG, fine features including cranial bones, spine, and dorsal fin are distinctly visualized. Even subtle differences between soft tissues and bones, as well as low-contrast tail fin structures, can be clearly distinguished, demonstrating the great potential of Ag-PG for biological imaging. In addition, radiation images recorded in Ag-PG can be stably stored at room temperature for more than 300 days and erased by annealing at 400 °C for 2 h, allowing repeated use of the material. A disk-type X-ray imaging detector composed of Ag-PG and a LiF film was developed by Kurobori et al. [43] for diagnostic dosimetry and radiotherapy. The detector exhibits high spatial resolution, a wide dynamic range of eight orders of magnitude, and non-destructive readout capability, enabling dose imaging from low to high radiation levels, especially for X-ray and γ-ray imaging in clinical and diagnostic scenarios. A two-dimensional reconstructed dose distribution can be obtained using this rotating disk detector (2400 rpm) under 3 Gy X-ray irradiation with the same mask [64]. By integrating images collected at different depths (surface, 100, 200, 300, and 400 μm) with a confocal detection system equipped with a 0.90 NA objective (working distance: 1 mm), the three-dimensional dose distribution stored in the transparent detector can be reconstructed. In terms of industrial non-destructive testing, Nanto et al. [65] realized X-ray visualization of internal structures in integrated circuits (ICs) based on the RPL effect of Ag-PG.

The X-ray imaging setup for Ag-PG involves X-rays passing through a square-hole metal mesh and irradiating the Ag-PG, which records the corresponding structural image [11]. The stored image is then read out by a fluorescence microscope under 355 nm UV excitation and detected by a CCD with a physical pixel size of 15.6 μm [11]. Post-irradiation imaging results and spatial resolution measurements indicate that Ag-PG achieves a resolution of 10 lp/mm [66]. Furthermore, the modulation transfer function (MTF) curve shows that the spatial frequency reaches 730 lp/mm at MTF = 0.2 [11]. To further verify its high-resolution performance, Ag-PG has been shown to realize submicron spatial resolution (~0.7 μm) [11], confirming its superior ability in high-resolution imaging and promising potential in precision medical imaging. In addition, Ag-PG exhibits excellent stability: X-ray image information can still be read repeatedly after 300 days without degradation in spatial resolution [11].

## 4. Conclusions

Ag-PG, as one part of RPL materials, displays outstanding competitiveness in high-precision radiation detection and high-resolution imaging. The phosphate network provides flexible coordination environments and abundant non-bridging oxygen sites, which favor the generation and stabilization of Ag^0^ and Ag^2+^ luminescence centers. Benefiting from its unique structure, Ag-PG achieves a wide linear dose–response range, high radiation sensitivity, submicron spatial resolution, and repeatable write-erase-rewrite performance through thermal annealing. These merits enable its practical applications in radiation dosimetry, 2D/3D dose mapping, biological imaging, and industrial non-destructive testing, filling the application gaps of traditional scintillators, OSL, and TSL materials in stable storage and non-destructive repeated readout.

To address these limitations, future research should focus on four key directions: (i) optimization of fabrication processes, including melt–quenching and atmosphere control, as well as the introduction of gradient annealing and flux doping to eliminate bubble defects, reduce internal stress, and improve yield and mechanical stability; (ii) matrix modification through metal oxide doping and network modifier engineering to enhance chemical durability and suppress Ag ion aggregation and signal degradation; (iii) advanced characterization using synchrotron radiation and time-resolved spectroscopy, combined with first-principles simulations, to elucidate the evolution of luminescence centers and RPL mechanisms; (iv) expansion toward application-oriented developments, including integration into miniaturized and flexible detector platforms; and (v) exploring composition tuning and multi-element co-doping strategies to further enhance radiation sensitivity, extend the linear dose range, and realize multi-type radiation discrimination. With continued advances, high-performance Ag-PG is expected to be further developed and widely applied in advanced medical imaging, micro/nanoscale radiation detection, and non-destructive testing in the nuclear industry, thereby promoting the evolution of radiation detection technologies toward higher precision, reusability, and visualization.

## Figures and Tables

**Table 1:** Excitation and emission peaks of Ag-PG.

Ag-PG	RPL Centre	λex/nm	λem/nm	c (Ag^+^)	Ref.
GD-450	Ag^0^, Ag^2+^, Ag_2_^+^ … Ag_m_^n+^	350	470 630	0.17 wt%	[41]
GD-450	Ag^0^, Ag^2+^	375 /	460 630	0.17 wt%	[42]
GD-450	Ag^0^, Ag^2+^	270/340 310	460 560	0.17 wt%	[43]
GD-450	Ag^0^, Ag^2+^	270 354	460 560	0.17 wt%	[44]
GD-302M	Ag^0^, Ag^2+^	349 /	400–500 600–700	0.17 wt%	[45]
FD-7	Ag^0^, Ag^2+^	/ 360/390	460 ± 35 650 ± 35	0.17 wt%	[46]
Na–Al/Ag	Ag^0^, Ag^2+^	/ 365	/ 635	0.1 wt%	[47]
K–Al/Ag	Ag^0^, Ag^2+^	/ 340	460 620	0.17 wt%	[48]
GD450/GD302M	Ag0, Ag^2+^, Ag_2_^+^	/ 350	450 650	0.17 wt%	[49]

**Table 2:** Decay time of Ag-PG.

Materials	RPL Centre	λem/nm	Decay Time	Ref.
Na–Al/Ag	Ag^0^ Ag^2+^	450 650	τ1 = 1.74 μs, τ2 = 19.74 μs, τ1 = 4.15 μs, τ2 = 13.09 μs	[54]
Na–Al/Ag	Ag^0^ Ag^2+^	/ 635	/ τ1 < 2 μs, τ2 > 20 μs	[22]
Na–Al/Ag	Ag^0^ Ag^2+^	460 ± 35 635 ± 35	<τ> = 4.5–30 × 10^−3^ μs, <τ> = 20 μs	[55]
Na–Al/Ag	Ag^0^ Ag^2+^, Ag_2_^+^	450 650	<τ> = 4.5 × 10^−3^ μs, <τ> = 2.3 μs	[46]
Na–Al/Ag	Ag^0^ Ag^2+^	450 650	<τ> = 25.29 μs, <τ> = 19.83 μs	[11]
Na–Al/Ag	Ag^0^ Ag^2+^	/ 635	/ τ1 < 2 μs,τ2 = 2–20 μs τ3 > 20 μs	[42]

## Data Availability

No new data were created or analyzed in this study. Data sharing is not applicable to this article.
